# Targeting EZH2 in Multiple Myeloma—Multifaceted Anti-Tumor Activity

**DOI:** 10.3390/epigenomes2030016

**Published:** 2018-09-03

**Authors:** Mohammad Alzrigat, Helena Jernberg-Wiklund, Jonathan D. Licht

**Affiliations:** 1 Division of Hematology and Oncology, Department of Medicine, University of Florida Health Cancer Center, University of Florida, Gainesville, FL 32610, USA; jdlicht@ufl.edu; 2 Science for Life Laboratory, Department of Immunology, Genetics and Pathology, Rudbeck Laboratory, Uppsala University, Uppsala, SE-75185 Uppsala, Sweden; helena.jernberg_wiklund@igp.uu.se

**Keywords:** epigenetics, EZH2, multiple myeloma, epigenetic therapy

## Abstract

The enhancer of zeste homolog 2 (EZH2) is the enzymatic subunit of the polycomb repressive complex 2 (PRC2) that exerts important functions during normal development as well as disease. PRC2 through EZH2 tri-methylates histone H3 lysine tail residue 27 (H3K27me3), a modification associated with repression of gene expression programs related to stem cell self-renewal, cell cycle, cell differentiation, and cellular transformation. EZH2 is deregulated and subjected to gain of function or loss of function mutations, and hence functions as an oncogene or tumor suppressor gene in a context-dependent manner. The development of highly selective inhibitors against the histone methyltransferase activity of EZH2 has also contributed to insight into the role of EZH2 and PRC2 in tumorigenesis, and their potential as therapeutic targets in cancer. EZH2 can function as an oncogene in multiple myeloma (MM) by repressing tumor suppressor genes that control apoptosis, cell cycle control and adhesion properties. Taken together these findings have raised the possibility that EZH2 inhibitors could be a useful therapeutic modality in MM alone or in combination with other targeted agents in MM. Therefore, we review the current knowledge on the regulation of EZH2 and its biological impact in MM, the anti-myeloma activity of EZH2 inhibitors and their potential as a targeted therapy in MM.

## 1. Introduction

Development and cell fate determination depend on intricate regulatory networks that control gene expression in a temporal, spatial and homeostatic manner. The term epigenetics was first coined in 1942 by Conrad Hal Waddington referring to phenotype changes that are not caused by changes in the genotype [1]. In current times, epigenetics is recognized as heritable changes in chromatin structure leading to various gene expression programs that are independent of the underlying DNA sequence. Epigenetic regulation of the genome has important roles in all aspects of organismal development (e.g., reproduction, cell proliferation and differentiation, tissue homeostasis, aging, and pathophysiology of diseases). At the molecular level, epigenetics describes the complex and dynamic interactions between the transcription factor repertoire and chromatin to build up chromatin states that control the accessibility of the underlying DNA and the information it contains to establish and maintain cellular identity required for the development of multicellular organisms like us [2,3]. DNA methylation, histone posttranslational modifications, non-coding RNA, chromatin remodeling and the nuclear 3-dimentional organization are all epigenetic mechanisms that control chromatin structure and DNA accessibility, thus defining cellular state and identity [2–5]. Aberrant changes in epigenetic mechanisms have thus been linked to a wide range of developmental defects and cell fate determination, as well as to diseases such as cancer [6,7].

Histones form a complex with the DNA making up the chromatin. Like all proteins, histones are subjected to various covalent chemical post-translational modifications (PTMs) at their N-terminal (tails) and globular domains, which in turn affect all chromatin templated processes (i.e., gene transcription and mRNA splicing, DNA replication, recombination and repair). Mechanistically, histone PTMs function either by disrupting chromatin contacts or by affecting the recruitment of non-histone proteins to chromatin, in turn thereby dictating the higher-order chromatin structure and orchestrating the ordered recruitment of protein complexes to manipulate the chromatin [8–10]. Histones can be modified by various modifications including acetylation, methylation, phosphorylation, ubiquitination, SUMOylation, ADP ribosylation, among others. Each of these modifications represent a code defining a certain chromatin state or structure. For example, histone lysine acetylation and phosphorylation are associated with open chromatin structure (euchromatin) i.e., regions of active gene transcription [11,12]. Histone lysine methylation is associated with a more complex regulatory output, which depends on the amino acid residue being methylated and the state of methylation (mono, di or tri-methylation). For example, methylation of histone 3 lysine 4 (H3K4me1 and H3K4me3) and methylation of histone 3 lysine 36 (H3K36me2 and H3K36me3) are associated with gene activation, while H3K9me3, H3K27me3 and H4K20me3 signals transcriptional repression [8,13,14]. Histone proteins are also subjected to methylation at arginine (R) residues, which affect high-order chromatin structure and the recruitment of protein complexes regulating chromatin-based processes. Like lysine methylation, arginine methylation has different regulatory meaning e.g., can activate or repress gene transcription depending on the methylated arginine residue and the state of methylation i.e., mono-methylation, symmetrical or asymmetrical di-methylation [15,16]. Furthermore, histone PTMs also show a substantial cross-regulation in the so-called histone code, leading to even more complex regulatory networks [17,18]. The levels and genome-wide distribution of histone marks are regulated by an intricate network of proteins that install the modifications “writers”, recognize them “readers”, and remove them “erasers”. Together this machinery provides the platform for targeting particular genomic sites ensuring proper biological outcomes [19,20]. Dysfunction or mutations in each of these proteins can thus lead to aberrant global or focal histone PTMs profiles and have been linked to tumorigenesis.

## 2. Deregulation of Chromatin Regulators in Multiple Myeloma

Multiple myeloma (MM) is a malignancy of antibody producing plasma cells (PCs) characterized by the accumulation of monoclonal PCs within the bone marrow (BM). MM as a tumor is derived from a premalignant benign phase known as monoclonal gammopathy of undetermined significance (MGUS). This MGUS state may progress into a premalignant and asymptomatic smoldering multiple myeloma (SMM), and finally to the symptomatic MM that becomes widely spread within the BM. MM can eventually in late stages, and after multiple therapies, develop into a disseminated form known as plasma cell leukemia (PCL) [21–23]. MM is a heterogeneous disease characterized by a complex genetic makeup [24–28], as well as diverse phenotypic symptoms [29–31], manifested as a patient-to-patient variation in tumor-clonal composition, disease management, overall survival and response to treatment. To identify early common tumor initiating events and to improve disease management and therapy, several international efforts have been initiated to stratify MM patients into distinct groups using genetic aberrations [32,33] and gene expression profiles [34,35]. Genetically, MM is divided into two major groups; hyperdiploid and non-hyperdiploid. The hyperdiploid group is characterized by trisomies of odd-numbered chromosomes and is associated with favorable prognosis, while the non-hyperdiploid group is well known to harbor translocations involve the immunoglobulin heavy chain (IgH), many of which are markers of poor prognosis in MM [23,36]. For example, chromosomal translocations t(4;14), t(14;16), and t(14;20) resulting in enhanced expression of *NSD2* (*MMSET/WHSC1*), *MAFC* and *MAFB* oncogenes, respectively, are major markers of poor prognosis in MM [37]. The stratification of MM in subgroups has contributed to a better understanding of MM biology, management and identification of novel treatment regimens that have improved MM patient survival up to 10 years in some cases [38–40]. The current treatment strategies are focused on killing the malignant PCs by induction of wide-range stress responses utilizing proteasome inhibitors (bortezomib) and histone deacetylase (HDAC) inhibitors (Valproic acid), or by more specific targeting agent such as immunomodulatory drugs (thalidomide and lenalidomide) to deprive the MM cells of key oncogenic transcription factors (e.g., Ikaros (IKZF1) and Aiolos (IKZF3) [36,41–43]). However, MM remains largely incurable due to the development of drug resistance and relapse, which urges the need to develop new therapeutic strategies that directly combat the malignant PCs, but also to reduce disease-associated pathologies such as bone resorption, kidney failure and immune deficiency.

In addition to the extensive genetic abnormalities characterizing the MM genome, aberrant epigenetic profiles have been suggested as important contributing factors in MM progression and resistance to therapy, as reviewed elsewhere [44–48]. Large scale analysis of MM genome in patients at diagnosis and relapse have identified epigenetic modifying enzymes, chromatic remodeling complexes and histone protein encoding genes as recurrently mutated in MM patients [42,49–52]. Recently, whole-exome sequencing analysis of 463 newly diagnosed MM patient (the UK NCRI Myeloma XI study-MyXI) revealed that mutations in epigenetic enzymes are common among MM patients i.e., 53% of the patients harbored epigenetic mutations, but the frequency of each epigenetic mutation in these patients is low ~2% [42]. Intriguingly, targeted sequencing of 156 previously relapsed cases at the University of Arkansas for Medical Sciences (UAMS) demonstrated an increase in the mutational frequency of some of these epigenetic mutations thus suggesting a role for epigenetic changes in MM progression [42]. For instance, mutations in *DNMT3A* and *TET2* increase from 0.4% and 1.1%, respectively in the MyXI to 5.1% and 2.6%, respectively in the UAMS. Also, there is an increase in the percentage of patients with mutations in the MLL histone methyltransferases family mainly *MLL2* (1.3% in MyXI to 3.9% in UAMS) and *MLL3* (1.5% in MyXI to 6.4% in UAMS). In addition, mutations in the *CREBBP* acetyltransferase increase in relapsed MM patients (0.7% in MyXI to 3.9 in UAMS) [42]. These findings require functional assays to fully unleash the impact of epigenetic mutations in MM biology.

In addition to genetic changes affecting epigenetic modifiers, deregulated expression of some epigenetic modifiers has been demonstrated in MM. For example, the polycomb group protein BMI-1 is overexpressed in MM and is required for MM cell growth in vitro and in vivo [53,54]. BMI-1 supports MM cell growth by inhibiting apoptosis through repression of the pro-apoptotic gene *BIM* [53]. High *BMI-1* expression levels are detected in patients at relapse and correlate with shorter overall survival in relapsed/refractory MM patients treated with bortezomib or dexamethasone [54]. The histone methyltransferase NSD2 is overexpressed in the t(4;14) patient subgroup, which represents 15–20% of MM patients and indicates poor prognosis [55,56]. NSD2 demonstrates oncogenic functions in MM by changing the chromatin landscape and gene expression profiles as well as increasing resistance to chemotherapy by enhancing DNA repair [57–59]. Cross-regulation between genetic lesions and aberrant epigenetic profiles such as DNA methylation [24,60], histone modifications [57,58] and non-coding RNA [61–63] have been documented to be of importance in the molecular pathogenesis of MM, and to be operational as predictors of prognosis and poor outcome of MM. Therefore, compounds or agents that target epigenetic mechanisms have been suggested as a promising therapeutic modality in MM [64–67]. This new strategy is currently under scrutiny by the recent Food and Drug Administration (FDA) approval of the pan-HDAC inhibitor panobinostat (PAN) in combination with bortezomib and dexamethasone as a third-line therapy in relapsed and/or refractory MM patients [68–70]. It is, however, important to state that the anti-MM effects of HDAC inhibitors are not solely mediated by chromatin and gene expression changes, rather it is likely the product of multiple underlying consequences including, but not limited to, apoptosis, autophagy, proteasome inhibition, protein recycling, suppression of angiogenesis and drug resistance.

## 3. EZH2 in Multiple Myeloma

The Enhancer of Zeste Homolog 2 (EZH2), is the enzymatic subunit of the polycomb repressive complex 2 (PRC2), an important regulator of normal development as well as disease. PRC2 via EZH2 installs the H3K27me3 repressive histone mark, which regulates expression programs related to stem cell self-renewal, cell cycle check-points and cellular differentiation, indicating that aberrant EZH2 activity may disturb normal development and tissue homeostasis leading to pathological consequences including cellular transformation [71–73]. Following the discovery that EZH2 functions as a chromatin modifying enzyme, a large number of reports have linked EZH2 to hallmarks of cancer via modulating the epigenome, leading to aberrant transcriptome in cancer cells making it a promising target for therapy [74–76]. The common findings are that EZH2 levels are deregulated in cancer tissues compared with corresponding normal tissues, and that high EZH2 levels correlate with advanced stages of disease and poor prognosis. Among these reports, altered EZH2 activity and levels have been most extensively documented in prostate, breast cancer, lymphoma, colon, myeloma, glioblastoma and medulloblastoma [77,78]. Additionally, EZH2 has been reported to be subjected to gain of function mutations that increase global levels of H3K27me3 in lymphoma and rare cases of melanoma, and lastly EZH2 inhibitors are in active clinical trial for lymphoma [79]. The consequences of EZH2 altered expression and activity are manifested as changes in the expression of genes that promote differentiation, restrain proliferation, enhance apoptosis and suppress invasion and metastasis. The role of EZH2 in cancer have been extensively reviewed elsewhere [77,78,80–83]. Herein we summarize the current knowledge on the role of EZH2 in multiple myeloma (MM).

**Figure 1.**
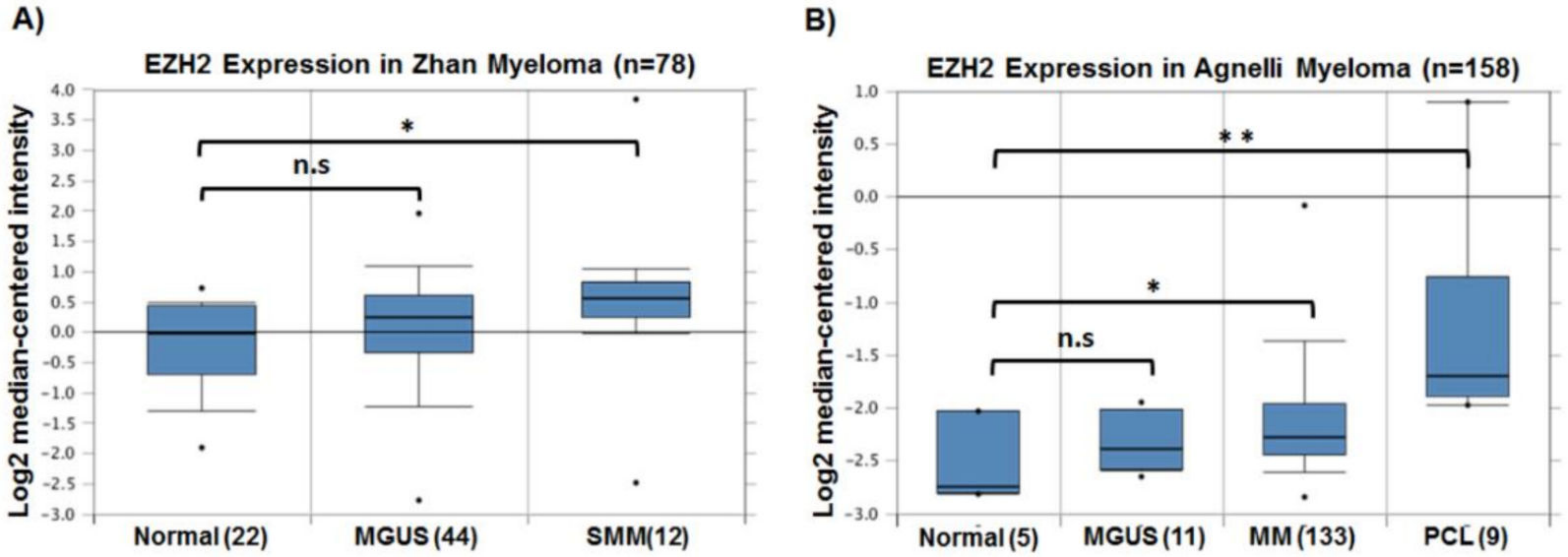
Enhancer of zeste homolog 2 (EZH2) expression increases during multiple myeloma (MM) progression. Analysis of EZH2 expression in two MM studies available in Oncomine database [103]. (**A**) Shows the increase in mean expression of EZH2 in smoldering multiple myeloma (SMM) compared with normal and monoclonal gammopathy of undetermined significance (MGUS) [104]. (**B**) Mean EZH2 expression is significantly higher in MM and plasma cell leukemia (PCL) patients compared to normal and MGUS [105]. The numbers in the brackets represent the number of patients in each category. n.s. *p* > 0.05, * *p*-value < 0.05; ** *p*-value < 0.01.

EZH2 is a common epigenetic enzyme that has been shown to be deregulated in MM. Initial global gene expression profiling in MM, MGUS and normal BM plasma cells has revealed EZH2 overexpression in an aggressive MM subgroup with a gene expression profile resembling that of human MM cell lines (HMCLs); a representative of the most advanced cases of MM [84,85]. Following this observation, Croonquist. et al. [86] showed that EZH2 expression in MM is driven by interleukin-6 (IL-6), an essential MM growth factor enriched in the BM milieu. This study showed that EZH2 overexpression is required for the growth of HMCLs and for inducing proliferation of HMCLs in an IL-6-independent manner. This was especially evident in HMCLs harboring mutations in K- and N-*RAS*, suggesting context-specific functions of EZH2 in MM [86]. Several gene expression studies demonstrated an increase in EZH2 expression during MM development from MGUS and SMM reaching its maximum at the PCL stage (Figure 1), suggesting disease progression-related functions of EZH2. It is worth mentioning that overexpression of other core subunits of the PRC2 complex; SUZ12 and EED have also been reported during MM progression, which may suggest that EZH2 functions in the context of the PRC2 complex in MM [87]. Recently, gene expression analysis of the UAMS (*n* = 1621) data set confirmed EZH2 overexpression in MM, especially in the proliferation subgroup (PR) and the 70-gene prognostic score (GEP70) group defining high-risk patients, proposing in this report that EZH2 expression may contribute to the high-risk phenotype in MM [88]. Moreover, EZH2 overexpression was suggested to be indicative of poor prognosis in MM i.e., shortened progression-free and overall survival, as well as reduced median overall survival based on multivariate analysis in two large independent data sets of phase III clinical trial patients, MyIX (*n* = 259) and UAMS-TT (*n* = 123) [88].

**Figure 2.**
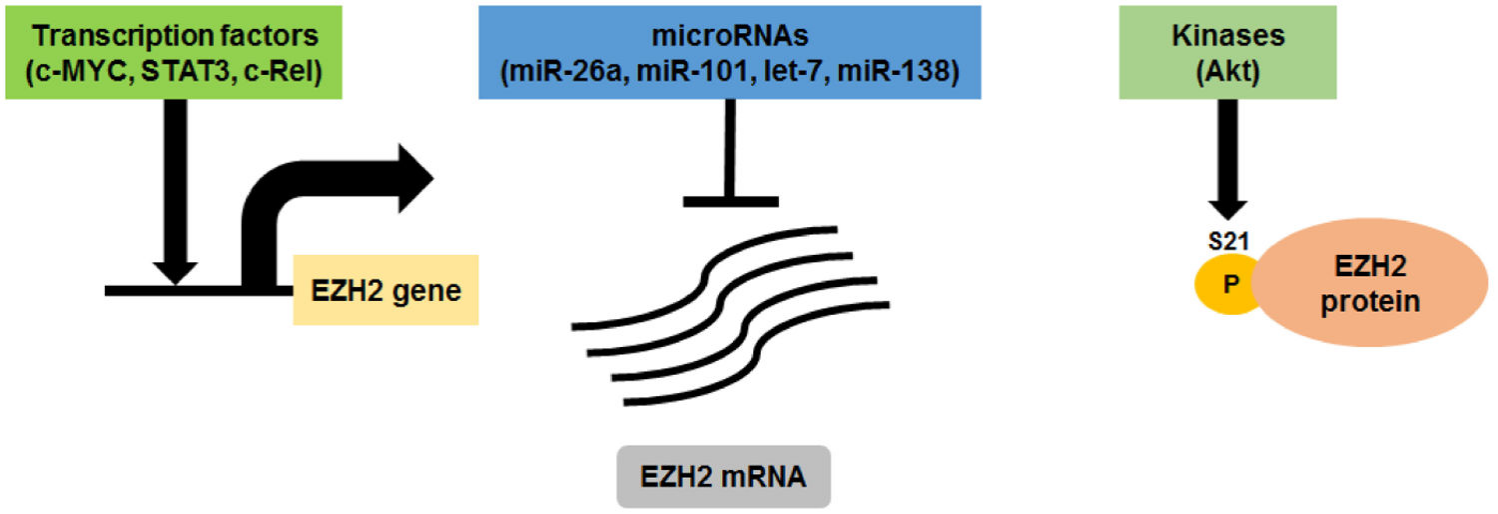
EZH2 expression and activity is regulated at multiple levels in MM. EZH2 expression is regulated by multiple essential oncogenic transcription factors and tumor suppressor microRNAs in MM. EZH2 enzymatic activity is regulated by Ser21 phosphorylation mediated by Akt kinase that function downstream of insulin-like growth factor-1 receptor (IGF-1R) and phosphoinositide 3 (PI3) kinase signaling pathways.

The aberrant expression and activity of EZH2 in MM is regulated at multiple levels as shown in Figure 2. For example, IL-6 signaling pathway, as well as key oncogenic transcription factors such as STAT3, c-MYC, and members of the NF-κB pathway, can directly drive EZH2 transcription during MM progression [86,89]. In addition, high EZH2 mRNA levels in MM can be linked to the downregulation of several microRNA species that are known to target EZH2 such as miR-26a, miR-101, let-7, and miR-138 [90–92]. More recently, the EZH2 methyltransferase activity is found to be inhibited by AKT-mediated phosphorylation at Serine 21 (S21) residue in drug-resistant MM cells that are in direct contact with bone marrow stromal cells [93]. The fact that EZH2 levels and activity are regulated at multiple layers suggests EZH2 as an important contributing factor in MM progression and the development of drug resistance. Despite that recent cancer sequencing projects have reported recurrent point mutations in *EZH2* in other hematopoietic tumors e.g., gain of function mutations in B-cell lymphomas [94–97] and loss of function mutations in T-cell acute lymphocytic leukemia [98], myelodysplastic syndromes, and myeloproliferative neoplasms [99–101], no mutations in *EZH2* have been identified in hundreds of MM patients whose tumors have been sequenced [42,102].

The fact that EZH2 has important roles in normal development as well as tumorigenesis, partly by regulating gene expression, has inspired several studies to define the genome wide-distribution of H3K27me3 and the nature of PRC2 targets in MM. Initially, genes in the lowest decile of expression in MM expression data sets were enriched in previously defined targets of PRC2/EZH2 in human embryonic fibroblasts [87]. Interestingly, these PRC2 targets are overrepresented among genes under-expressed in MGUS and MM patients and strongly correlated with decreased expression in International Staging System (ISS) stage III MM, compared to stage I and II [87]. In addition, genome-wide profiling of H3K27me3 mark combined with RNA-Seq in MM patients suggest PRC2-mediated gene silencing as a mechanism of gene repression during MM progression [102]. Notably, the MM H3K27me3 epigenetic profile correlates with poor survival [102]. These studies as well as the MyIX and UAMS studies suggest an important role for EZH2 and its targets gene in MM progression and might serve as a poor prognostic marker in MM.

EZH2 overexpression in MM is not generally associated with a global increase in histone methylation and presumably functions focally at specific target genes. By contrast, in t(4;14)-positive MM the enhanced expression of the H3K36 methyltransferase NSD2 changes the balance between the H3K26me2 and H3K27me3 resulting in increased levels of H3K36me2 and a striking decrease in H3K27me3 levels [57,58] NSD2 functions as an oncogene in MM and its overexpression restored the tumorigenicity of t(4;14)-negative MM cells. This is associated with increased transcriptional activity of oncogenic programs that are dependent on H3K36me2 chromatin mark [106]. Despite the decrease in H3K27me3 in NSD2 overexpressing cells, H3K27me3 and EZH2 are enriched at specific genomic loci harboring genes known to play roles in normal germinal center B-cells as well as genes known to be targets of c-*MYC* oncogene in B-cells [58]. Interestingly, EZH2 and H3K27me3 demarcated regions were found to be over-represented in CCCTC-binding factor (CTCF) binding sites [58]. CTCF is known insulator that blocks the spread of chromatin marks, which may suggest that EZH2 cooperates with NSD2 and H3K36me2 to establish chromatin boundaries and structures defining the t(4;14) subset of MM. Accordingly, t(4;14) NSD2 overexpressing MM cells were more sensitive to an EZH2 inhibitor than an isogenic cell line without NSD2 expression. This suggests that EZH2 may demonstrate context-dependent oncogenic activities in MM. Further supporting this notion, Ezponda et al. [107] demonstrated increased sensitivity to EZH2 inhibition in MM cells harboring KMD6A/UTX deletion, when compared to MM cells expressing wild-type KDM6A/UTX.

## 4. EZH2 Inhibition in Multiple Myeloma

**Figure 3.**
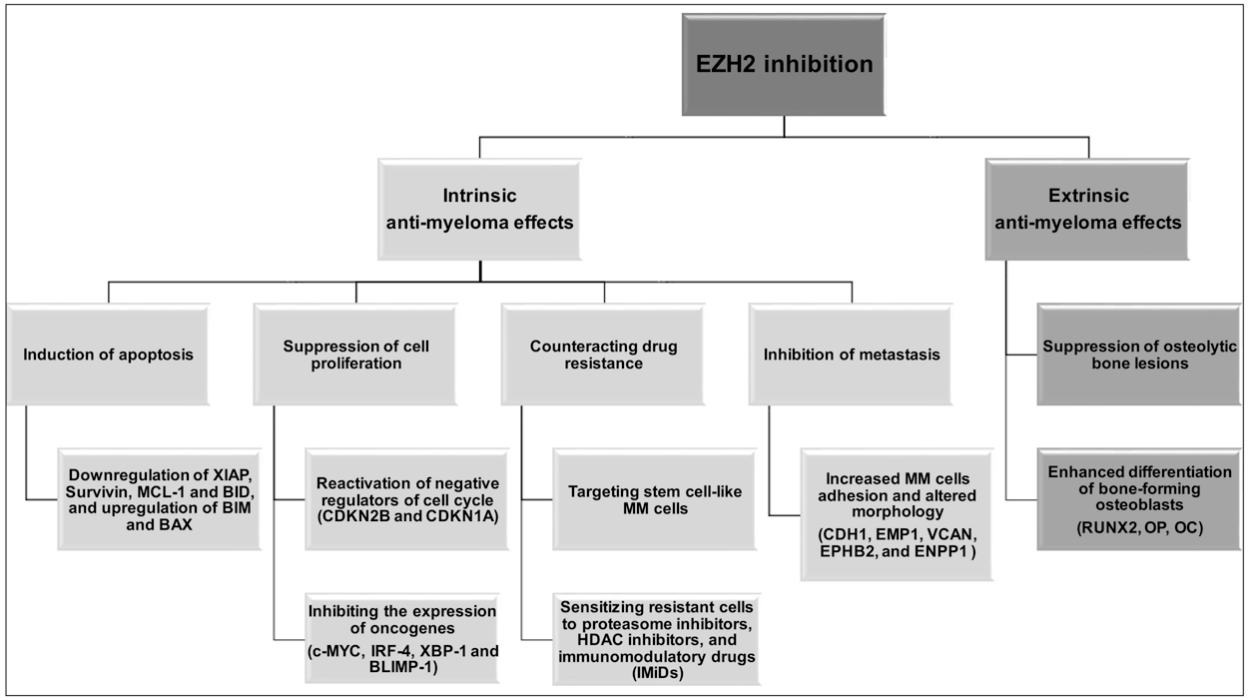
EZH2 inhibitors demonstrate multifaceted anti-myeloma activity by affecting the malignant plasma cells and the bone marrow microenvironment. XIAP: X-linked inhibitor of apoptosis; MCL1: MCL1, BCL2 family apoptosis regulator; BID: BH3 interacting domain death agonist; BIM: also known as BCL2L11 (BCL2 like 11). BAX: BCL2 associated X, apoptosis regulator. CDKN2B: cyclin dependent kinase inhibitor 2B; CDKN1A: cyclin dependent kinase inhibitor 1A; IRF4: interferon regulatory factor 4; XBP1 X-box binding protein 1; BLIMP-1: also known as PRDM1 (PR/SET domain 1); CDH1: Cadherin 1; EMP1: Epithelial membrane protein 1; VCAN: Versican; EPHB2: Ephrin receptor B2; ENPP1: Ectonucleotide pyrophosphatase/phosphodiesterase 1; RUNX2: Runt related transcription factor 2; OP: *Osteopontin*; *OC*: *Osteocalcin*.

Considering the nature of epigenetic processes being reversible and amenable to altered programing, epigenetic modifiers are especially interesting as targets for therapy in cancer. Many agents have been developed to target epigenetic processes including inhibitors of histone acetylation, histone methylation and demethylation as well as DNA methylation, with DNA methylation and histone deacetylation inhibitors entering clinical use [7,108]. EZH2 and/or PRC2 have been the focus for targeted inhibition due to their documented altered activity and levels in several tumor types including prostate, glioblastoma, B-cell lymphoma and multiple myeloma [77,78] and the suggestion that the associated H3K27me3 profile may confer stemness properties not only in human embryonic stem cells, but also in cancer cells. In 2010, we showed that EZH2/PRC2 inhibition using the broadly acting S-adenosylhomocysteine hydrolase inhibitor 3-Deazaneplanocin (DZNep) and the histone deacetylase inhibitor LBH589 (Panobinostat) depletes EZH2 and other PRC2 components from cells and demonstrated anti-MM effects using HMCLs in vitro and in the 5T33MM in vivo models [87]. LBH589, an HDAC inhibitor indirectly causes degradation of EZH2 through interference with protein chaperone function and reactivated the expression of genes repressed by PRC2 in vitro and in vivo, reduced tumor load, and increased overall survival [87]. Nonetheless, DZNep and LBH589 are non-specific inhibitors of PRC2 and other studies showed that anti-myeloma activity could be attributed to non-PRC2 mediated mechanisms including the generation of reactive oxygen species (ROS) response, signaling pathways and protein stability perturbations [65,109–111]. The recent development of highly potent small-molecule inhibitors of EZH2 have opened new avenues to evaluate the therapeutic potential of EZH2 in tumors dependent on EZH2 enzymatic activity. Recently, several reports have documented the potential use of several EZH2 specific inhibitors as anti-MM agents (Table 1). All these studies demonstrated multifaceted anti-MM activity of EZH2 inhibitors by affecting intrinsic (within the MM cell) and extrinsic (affecting the BM microenvironment) oncogenic pathways promoting the growth and survival of MM cells (Figure 3), which highly suggest EZH2 as promising target for therapy in MM.

We and others have investigated the anti-myeloma activity of EZH2 inhibitors. For instance, EZH2 inhibition by GSK343 and UNC1999 demonstrates anti-myeloma activity by reducing the survival of MM cell lines and CD138^+^ MM cells isolated from newly diagnosed patients [58,102,112]. The inhibitors of EZH2 reduced the global levels of H3K27me3 mark and reactivated the expression of genes involved in apoptosis, cell differentiation, senescence and autophagy. Notably, EZH2 inhibition results in the downregulation of essential MM oncogenic transcription factors such c-*MYC*, *IRF-4*, *XBP-1* and *BLIMP-1* [58,102,112]. The anti-myeloma activity of EZH2 inhibitors was further supported by Hernando et al. using the EZH2 inhibitor EPZ-7438 in vitro and in vivo xenograft model [117]. Specifically, the authors found that EZH2 inhibition induces the expression of epithelial tumor suppressor genes including *CDH1*, *EMP1*, *VCAN*, *EPHB2*, and *ENPP1*, which enhance MM cells adhesion properties [117]. These data may suggest an important role for EZH2 in promoting bone marrow dissemination of the tumor during disease progression by repressing the epithelial tumor suppressor gene signature. In another study, EZH2 inhibition by using GSK126 induces apoptosis in MM and was here proposed to eradicate the stem-cell like MM cells by blocking the Wnt/β-catenin pathway [114]. The fact that MM EZH2/H3K27me3 targets are enriched with targets of polycomb in human embryonic stem cells further supports the notion that EZH2 inhibitors might target stem-cell like MM cells, which needs to be further explored. Furthermore, EZH2 inhibition with EPZ005687 demonstrates anti-MM effects via the upregulation of cell cycle negative regulators e.g., *CDKN2B* and *CDKN1A* leading to cell cycle arrest followed by apoptosis [88]. In addition to the reactivation of mRNAs encoding proteins with tumor suppressor functions, EZH2 inhibition induces the expression of tumor suppressor microRNA species that are predicted or documented to negatively regulate the mRNA levels of essential MM oncogenes such as c-MYC [58,112,120], or induce drug resistance [92]. Interestingly, even though all these studies have demonstrated anti-MM effects of EZH2 inhibitors, considerable variation in terms of the reactivated genes is observed. This could be due to the great genetic heterogeneity of HMCLs, variation of cell lines used in each study, duration of treatment i.e., short vs long term exposure to EZH2 inhibitors, and the use of growth factors e.g., IL-6 to support the growth of HMCLs. Nevertheless, all studies concluded that cell cycle arrest, apoptosis, and downregulation of c-MYC signature are common consequences of EZH2 inhibition in MM. It should be noted that response to EZH2 inhibitors is not universal among HMCLs in these studies, despite consistent global H3K27 demethylation. The presence of other genetic lesions in the HMCLs may obviate a dependence on EZH2 function, for example, MYC translocations might alter the usual control of c-MYC expression or alternatively EZH2 expression may support gene regulation independent of its methyltransferase activity. To address this question would require comparison of the effects of knockdown or knockout of EZH2 and other PRC2 components in a panel of well-characterized HMCLs to the effects of EZH2 inhibitors.

In addition to their anti-myeloma activity as single agents, EZH2 inhibitors may be effective anti-myeloma agents in combination with clinically relevant myeloma regimens. Analysis of gene expression profiles of pretreatment samples from multiple myeloma patients enrolled on the APEX039 clinical study revealed that high levels of *EZH2* expression is indicative of poor response to bortezomib treatment [113]. Similarly, ectopic expression of EZH2 in HMCLs conferred resistance to bortezomib treatment [113]. Following this line of reasoning, EZH2 inhibition utilizing UNC1999 was shown to overcome drug resistance and enhance the anti-MM activity of bortezomib and carfilzomib in resistant cell lines and CD138^+^ plasma cells isolated from MM patients [113]. The authors suggested the tumor suppressor *NR4A1* as candidate gene defining MM sensitivity to bortezomib by directly regulating the expression of *c-MYC* oncogene. Bortezomib resistant HMCLs and MM patients have lower *NR4A1* expression, which correlate with high expression of c-MYC and its target genes [113]. Notably, UNC1999-mediated inhibition of EZH2 depleted the *NR4A1* promoter of the H3K27me3 mark and induced *NR4A1* expression leading to the suppression of c-MYC. These observations are enhanced by combination treatment of UNC1999 and bortezomib [113]. Another EZH2 inhibitor, GSK126 also demonstrated synergistic anti-MM effects with bortezomib by promoting cell death [114], in part due to a marked decrease in the anti-apoptotic protein MCL-1 [114]. The contribution of EZH2 to bortezomib resistance in MM is further supported by Rastgoo et al. [92] through direct repression of the EZH2-targeting miR-138 and the tumor suppressor RNA-binding protein with multiple splicing (*RBPMS*) gene [92]. EZH2 inhibition using EPZ-6438 or miR-138 mimics reduced the H3K27me3 mark at the *RBPMS* promoter thus enhancing its expression [92]. RBPMS exerts its antitumor activity by inhibiting oncogenes such as *c-MYC* and the anti-apoptotic Bcl-2 protein or activating negative regulators of cell cycle such as p15INK4b, p21CIP1/WAF1, and p57KIP2 [121,122]. Collectively, these data may suggest EZH2 as mediator of resistance to proteasome inhibition in multiple myeloma and highlight that the combination of EZH2 and proteasome inhibitors might be useful in both newly diagnosed as well as proteasome inhibitor refractory MM patients.

An interesting report shows that a combinatorial inhibition of EZH2 and DNA methylation re-sensitizes immunomodulatory drug (IMiD)-resistant MM cells to lenalidomide or pomalidomide treatment [116]. IMiD-resistant MM cells are characterized by global changes in DNA methylation profile and reduced chromatin accessibility leading to prominent gene downregulation [116]. Interestingly, the gene repressive nature of IMiD-resistant MM cells did not affect the cereblon (CRBN) or other molecules involved in the CRBN pathway including IKZF1, IKZF3, IRF4 since their expression did not change with or without epigenetic sensitization. However, the study identified *SMAD3*, a transcriptional regulator and a core component of the canonical transforming growth factor beta (TGF-β) signaling pathway, as a commonly downregulated gene in all IMiD-resistant cell line utilized in the study [116]. SMAD3 expression has been shown to regulate the switch TGF-β signaling pathway between tumor suppressive or oncogenic effects in cancer. High SMAD3 levels are required for the tumor suppressive effects of TGF-β, while lower expression levels correlate with the tumor-promoting effect of TGF-β [123]. Whether IMiD-resistant myeloma cells utilize EZH2 to manipulate the TGF-β functions by regulating SMAD3 expression to gain proliferative advantage over the antitumor effects is an important concept. Further investigation is demanded, however, to fully understand the role of EZH2 in myeloma resistance to IMiDs. More recently, EZH2 inhibition as single treatment demonstrated anti-myeloma activity only in a subset of HMCLs despite the global decrease in H3K27me3 levels. However, pre-treatment of HMCLs with the EZH2 inhibitors EPZ-7438 and GSK126 enhanced the sensitivity of HMCLs to the FDA approved pan-HDAC inhibitor panobinostat irrespective of single agent EZH2 inhibitor sensitivity [115]. The later study again suggests that combinations of epigenetic inhibitors should be considered in novel anti-myeloma treatment. In support of this notion, the combination of EZH2 and BMI-1 (PRC1) inhibitors have synergistic anti-myeloma activity using HMCLs and CD138^+^ myeloma cells isolated from newly diagnosed or relapsed MM patients [124]. The use of EZH2 inhibitors as well as other epigenetic inhibitors to sensitize drug resistance to clinically relevant treatment protocols may suggest epigenetic changes as possible underlying mechanism contributing to drug resistance in MM. Importantly, this notion may provide a therapeutic advantage for MM patients with relapsed/refractory disease and should be further investigated in pre-clinical models as well as in clinical trial.

In addition to its intrinsic role in MM cells, two recent reports documented the role of EZH2 in modulating the BM microenvironment by regulating osteogenic differentiation of BM-derived mesenchymal stem cells (MSC) [125,126]. EZH2 represses osteogenic differentiation of MSC through direct regulation of runt related transcription factor 2 (*RUNX2*), *osteopontin (OP) and osteocalcin (OC)*, which are key transcription factors driving MSC osteogenic differentiation [125,126]. Activation of these genes during osteogenesis is regulated by an epigenetic switch at their promoters i.e., removal of H3K27me3 and the addition of H3K4me3. Interestingly, this chromatin switch is dependent on the recruitment of UTX/KDM6A-containing complex to counteract EHZ2 repression [125,126]. The authors revealed that EZH2 promotes adipogenesis on favor of osteogenesis, while UTX/KDM6A enhances osteogenesis and represses adipogenesis [126]. In MM pathology, MM cells induce the expression of the transcription repressor GFI1 in osteoblast precursors, which represses *RUNX2* expression resulting in osteoblast-differentiation blockade [127]. GFI1-mediated repression of *RUNX2* was shown to be dependent on the recruitment of HDAC1, LSD1, and EZH2 converting *RUNX2* promoter from a bivalent (H3K27me3/H3K4me3) state into a repressed state [128]. Notably, the EZH2 specific inhibitor GSK126 as well as the HDAC1 inhibitor MC1294 reversed the repressive chromatin architecture at *Runx2* promoter and thereby rescued osteoblast differentiation in osteoblast precursors exposed to MM cells in vitro or in osteoblast precursors from MM patients [128]. The contribution of EZH2 to the aberrant epigenetic switch affecting the composition of BM microenvironment leading to osteolytic bone destruction, a major contributor to MM patient morbidity and mortality, highly suggests EZH2 inhibition as a promising therapy in MM.

## 5. Conclusions

Deregulation in EZH2 expression and activity is evident in various types of tumors including MM. Given that EZH2 is overexpressed in MM and of prime importance in multiple oncogenic pathways promoting MM cell growth, survival and resistance to currently used treatments, supports its evaluation for use in targeted therapy in MM. Despite an initial phase I clinical trial (NCT02082977) using EZH2 inhibitors as single agent treatment has shown insufficient evidence of clinical activity, the use of EZH2 inhibitors in combination with current treatment protocols such as proteasome inhibitors, IMiDs, and dexamethasone may provide a therapeutic value for MM patients, especially for relapsed/refractory groups of MM patients. Furthermore, the identification of certain genetic defects in MM patients such as t(4;14) and UTX/KDM6A-deletion that pre-dispose to EZH2 inhibition may provide more effective and personalized treatment by using EZH2 inhibitors. Moreover, the identification of posttranslational modification that modulate EZH2 enzymatic activity e.g., Ser 21 phosphorylation and non-histone targets of EZH2 such as proliferating cell nuclear antigen (PCNA) may open new avenues to understand the molecular functions of EZH2 and the impact of EZH2 inhibitors in cancer therapy.

## Figures and Tables

**Table 1.** Summary of EZH2 inhibitors that have been tested for anti-myeloma activity.

EZH2 Inhibitor	Anti-MM Activity	Treatment Type	Reference
UNC1999	In vitro and in vivo	Single agent treatment or in combination with Bortezomib	[102,112,113]
GSK343	In vitro	Single agent treatment	[58,102,107]
GSK126	In vitro and in vivo	Single agent treatment or in combination with Bortezomib and Panobinostat	[107,114,115]
			
EPZ-7438	In vitro and in vivo	Single agent treatment or in combination with Lenalidomide, Pomalidomide, Bortezomib and Panobinostat	[115–117]
			
EPZ005687	In vitro	Single agent treatment	[88]
OR-S1 and OR-S2	In vitro	Single agent treatment	[118]
GSK2816126	Phase I clinical trial (NCT02082977)–Terminated	Single agent treatment	[119]
